# Supplementary material for: Linkage Disequilibrium Decay in Selected Cattle Breeds
Source: Animals (Basel). 2024 Nov 18;14(22):3317. doi: 10.3390/ani14223317 (PMC11590911; doi:10.3390/ani14223317)
Supplement: Supplementary file 1 [file animals-14-03317-s001.zip › Table S1.pdf]

**Table S1**. Quality score and percentage of 10 randomly chosen samples.

| Sample number (randomly chosen cattle) | Quality Score of sequences | Q20(%) | Q30(%) |
|---|---|---|---|
| 1 | 0.88 | 94.32 | 90.21 |
| 2 | 0.91 | 94.62 | 90.53 |
| 3 | 0.86 | 95.88 | 91.14 |
| 4 | 0.92 | 96.92 | 91.83 |
| 5 | 0.96 | 96.18 | 92.16 |
| 6 | 0.95 | 96.23 | 93.12 |
| 7 | 0.89 | 96.17 | 93.34 |
| 8 | 0.83 | 97. 91 | 93.82 |
| 9 | 0.94 | 95.14 | 92.18 |
| 10 | 0.94 | 94.72 | 90.34 |
