# Supplementary material for: Linkage Disequilibrium Decay in Selected Cattle Breeds
Source: Animals (Basel). 2024 Nov 18;14(22):3317. doi: 10.3390/ani14223317 (PMC11590911; doi:10.3390/ani14223317)
Supplement: Supplementary file 1 [file animals-14-03317-s001.zip › Table S2.pdf]

Table S2. Average D and standard deviations based on total SNPs for each autosomal chromosome in genome

Chromosome number	Breed				
	Sistani	Sahiwal	Nellore	Holstein	Hereford
BTA1	0.016±0.036	0.014±0.030	0.016±0.041	0.020±0.043	0.017±0.042
BTA2	0.023±0.042	0.010±0.020	0.015±0.033	0.019±0.046	0.021±0.048
BTA3	0.016±0.031	0.006±0.010	0.013±0.023	0.016±0.045	0.020±0.042
BTA4	0.028±0.062	0.014±0.024	0.006±0.015	0.012±0.032	0.021±0.048
BTA5	0.029±0.061	0.008±0.012	0.013±0.035	0.015±0.026	0.015±0.038
BTA6	0.018±0.032	0.013±0.023	0.017±0.034	0.014±0.023	0.013±0.034
BTA7	0.023±0.046	0.007±0.013	0.013±0.026	0.018±0.031	0.023±0.046
BTA8	0.019±0.043	0.018±0.033	0.023±0.038	0.029±0.058	0.023+0.050
BTA9	0.020±0.045	0.012±0.023	0.016±0.031	0.021±0.039	0.023±0.049
BTA10	0.013±0.030	0.014±0.021	0.019±0.040	0.014±0.025	0.023±0.054
BTA11	0.014±0.031	0.005±0.012	0.013±0.024	0.014±0.025	0.014±0.032
BTA12	0.025±0.059	0.011±0.026	0.07±0.023	0.005±0.010	0.030±0.059
BTA13	0.029±0.061	0.013±0.018	0.05±0.020	0.016±0.039	0.027±0.055
BTA14	0.014±0.030	0.012±0.022	0.013±0.029	0.017±0.019	0.019±0.039
BTA15	0.019±0.034	0.009±0.018	0.014±0.026	0.015±0.022	0.017±0.029
BTA16	0.012±0.032	0.007±0.023	0.011±0.019	0.014±0.042	0.020±0.045
BTA17	0.014±0.034	0.013±0.024	0.005±0.014	0.006±0.018	0.033±0.065
BTA18	0.019±0.043	0.011±0.028	0.010±0.024	0.009±0.012	0.029±0.049
BTA19	0.036±0.083	0.014±0.031	0.010±0.021	0.015±0.032	0.018±0.042
BTA20	0.024±0.039	0.012±0.021	0.09±0.023	0.011±0.035	0.035±0.073
BTA21	0.016±0.030	0.006±0.015	0.018±0.041	0.012±0.029	0.022±0.045
BTA22	0.022±0.035	0.007±0.011	0.014±0.030	0.010±0.023	0.017±0.036
BTA23	0.025±0.043	0.013±0.022	0.013±0.022	0.007±0.007	0.022±0.053
BTA24	0.026±0.044	0.004±0.019	0.017±0.035	0.012±0.026	0.020±0.048
BTA25	0.032±0.050	0.004±0.008	0.005±0.016	0.013±0.030	0.003±0.009

BTA26	0.021±0.037	0.017±0.041	0.003±0.009	0.023±0.043	0.023±0.056
BTA27	0.014±0.025	0.012±0.034	0.006±0.012	0.021±0.041	0.001±0.005
BTA28	0.017±0.030	0.015±0.035	0.004±0.010	0.004±0.011	0.017±0.035
BTA29	0.022±0.040	0.008±0.016	0.037±0.075	0.012±0.024	0.0178±0.042
