# Supplementary material for: Linkage Disequilibrium Decay in Selected Cattle Breeds
Source: Animals (Basel). 2024 Nov 18;14(22):3317. doi: 10.3390/ani14223317 (PMC11590911; doi:10.3390/ani14223317)
Supplement: Supplementary file 1 [file animals-14-03317-s001.zip › Table S3.pdf]

Table S3. Average D' and standard deviations based on total SNPs for each autosomal chromosome in genome.

Chromosome number	Breed				
	Sistani	Sahiwal	Nellore	Holstein	Hereford
BTA1	0.62± 0.56	0.65±0.59	0.66±0.55	0.66±0.48	0.69±0.42
BTA2	0.65±0.58	0.64±0.52	0.64±0.57	0.57±0.47	0.57±0.42
BTA3	0.60±0.43	0.63±0.50	0.58±0.45	0.54±0.40	0.67±0.47
BTA4	0.64±0.53	0.61±0.46	0.57±0.42	0.52±0.35	0.56±0.39
BTA5	0.61±0.53	0.59±0.39	0.60±0.42	0.54±0.41	0.53±0.40
BTA6	0.57±0.41	0.56±0.45	0.61±0.46	0.54±0.39	0.57±0.43
BTA7	0.63±0.55	0.61±0.43	0.65±0.45	0.60±0.40	0.53±0.38
BTA8	0.62±0.46	0.63±0.40	0.65±0.59	0.54±0.39	0.59±0.45
BTA9	0.59±0.40	0.60±0.41	0.60±0.45	0.57±0.42	0.45±0.35
BTA10	0.64±0.55	0.63±0.52	0.60±0.41	0.53±0.43	0.60±0.50
BTA11	0.54±0.40	0.60±0.52	0.58±0.49	0.52±0.36	0.52±0.40
BTA12	0.65±0.57	0.64±0.55	0.66±0.56	0.53±0.35	0.54±0.37
BTA13	0.63±0.50	0.60±0.46	0.56±0.37	0.57±0.40	0.58±0.40
BTA14	0.57±0.47	0.62±0.45	0.60±0.35	0.51±0.30	0.54±0.39
BTA15	0.63±0.55	0.60±0.50	0.57±0.46	0.54±0.44	0.56±0.48
BTA16	0.61±0.45	0.59±0.40	0.63±0.48	0.56±0.45	0.61±0.46
BTA17	0.64±0.55	0.61±0.50	0.61±0.49	0.51±0.35	0.60±0.50
BTA18	0.61±0.49	0.61±0.49	0.65±0.54	0.51±0.40	0.57±0.42
BTA19	0.64±0.58	0.65±0.62	0.65±0.55	0.58±0.40	0.55±0.45
BTA20	0.57±0.43	0.64±0.45	0.63±0.52	0.55±0.38	0.57±0.42
BTA21	0.62±0.48	0.60±0.42	0.54±0.40	0.53±0.65	0.61±0.49
BTA22	0.60±0.45	0.59±0.40	0.61±0.55	0.57±0.38	0.57±0.42
BTA23	0.63±0.51	0.55±0.45	0.63±0.53	0.54±0.47	0.56±0.47
BTA24	0.61±0.51	0.61±0.68	0.61±0.58	0.57±0.49	0.59±0.52
BTA25	0.60±0.48	0.60±0.55	0.62±0.57	0.50±0.48	0.53±0.53
BTA26	0.59±0.45	0.60±0.50	0.65±0.52	0.50±0.35	0.58±0.46

BTA27	0.57±0.43	0.57±0.47	0.59±0.52	0.62±0.40	0.54±0.43
BTA28	0.62±0.50	0.59±0.46	0.56±0.47	0.50±0.32	0.55±0.42
BTA29	0.60±0.44	0.64±0.48	0.63±0.49	0.62±0.42	0.63±0.40
